# Nanomaterials in Dentistry: Current Applications and Future Scope

**DOI:** 10.3390/nano12101676

**Published:** 2022-05-14

**Authors:** Pavan Kumar Pavagada Sreenivasalu, Chander Parkash Dora, Rajan Swami, Veeriah Chowdary Jasthi, Predeepkumar Narayanappa Shiroorkar, Sreeharsha Nagaraja, Syed Mohammed Basheeruddin Asdaq, Md. Khalid Anwer

**Affiliations:** 1 Department of Restorative Dentistry and Endodontics, College of Dentistry, King Faisal University, Al-Ahsa 31982, Saudi Arabia; psreenivasalu@kfu.edu.sa; 2 Department of Pharmaceutics, Maharshi Markendeshwar College of Pharmacy, Maharishi Markandeshwar Univerisity, Mullana, Ambala 133207, Haryana, India; chanddora@gmail.com; 3 Department of Oral and Maxillofacial Surgery and Diagnostic Sciences, College of Dentistry, King Faisal University, Al-Ahsa 31982, Saudi Arabia; vjasthi@kfu.edu.sa; 4 Department of Basic Medical Sciences, College of Medicine, King Faisal University, Al-Ahsa 31982, Saudi Arabia; pshiroorkar@kfu.edu.sa; 5 Department of Pharmaceutical Sciences, College of Clinical Pharmacy, King Faisal University, Al-Ahsa 31982, Saudi Arabia; 6 Department of Pharmaceutics, VidyaSiri College of Pharmacy, Off Sarjapura Road, Bangalore 560035, Karnataka, India; 7 Department of Pharmacy Practice, College of Pharmacy, AlMaarefa University, Dariyah, Riyadh 13713, Saudi Arabia; sasdaq@gmail.com; 8 Department of Pharmaceutics, College of Pharmacy, Prince Sattam Bin Abdulaziz University, Al-Kharj 11942, Saudi Arabia; m.anwer@psau.edu.sa

**Keywords:** nanoparticle, nanodentistry, nano-implant, dental

## Abstract

Nanotechnology utilizes the mechanics to control the size and morphology of the particles in the required nano range for accomplishing the intended purposes. There was a time when it was predominantly applied only to the fields of matter physics or chemical engineering, but with time, biological scientists recognized its vast benefits and explored the advantages in their respective fields. This extension of nanotechnology in the field of dentistry is termed ‘Nanodentistry.’ It is revolutionizing every aspect of dentistry. It consists of therapeutic and diagnostic tools and supportive aids to maintain oral hygiene with the help of nanomaterials. Research in nanodentistry is evolving holistically but slowly with the advanced finding of symbiotic use of novel polymers, natural polymers, metals, minerals, and drugs. These materials, in association with nanotechnology, further assist in exploring the usage of nano dental adducts in prosthodontic, regeneration, orthodontic, etc. Moreover, drug release cargo abilities of the nano dental adduct provide an extra edge to dentistry over their conventional counterparts. Nano dentistry has expanded to every single branch of dentistry. In the present review, we will present a holistic view of the recent advances in the field of nanodentistry. The later part of the review compiled the ethical and regulatory challenges in the commercialization of the nanodentistry. This review tracks the advancement in nano dentistry in different but important domains of dentistry.

## 1. Introduction

Nanotechnology has traveled a long way from a mere theoretical concept presented by Richard Feynman to the incorporation of nanotechnology in every aspect of life, such as dentistry [1]. Conventional dental technology has been enriched by the revolutionized principles of nanotechnology [2]. These principles drive a paradigm shift in the research outlook of nanotechnologists working in different dentistry domains, such as prosthodontics and orthodontics. Primarily, dental practitioners focus on the prophylactic treatment of dental caries and periodontal disease. Though at the time when tooth or periodontium suffer from infectious disease, the foremost aim of the treatment is to eradicate the pathogens, remove decayed tissues and restore them, providing long-lasting functionality in the oral cavity. Different approaches like top-down, bottoms-up, biomimetic, and functional approaches were explored for each aspect. 

Exquisite oral health implicates proper nutrition, which is associated with better quality of life. Undoubtedly teeth are a perpetual part of oral health [3]. Developing biocompatible nano products requires a sharp assessment of the anatomy and physiology of the target site, i.e., teeth and oral cavity. The close contact of nanomaterials with nanoparticles dictates the nanomaterial’s fate in the body. The anatomy, structure, and chemical composition of human tooth tissues are diversified and the understanding of each part and individual characteristic abundance of material in each layer provide insight into how the nanomaterial should be formed specifically as per the requirement of the target layer. From outside to inside, the tooth can be divided majorly into Enamel, Dentin and Pulp, Cementum, and Periodontal ligament [4]. Tooth enamel is the most mineralized and hard solid crystalline structure of hydroxyapatite (HA) with immensely strong intermolecular forces. Composition vise it is 96 wt.% inorganic material and 4 wt.% organic material and water. Whereas around 70% of the inorganic material is evident in dentin. It consists of HA biocomposites that encircle the collagen fibers, the reason why HA is the most widely used material for dental products, especially in nanoproducts [5].

**Figure 1:**
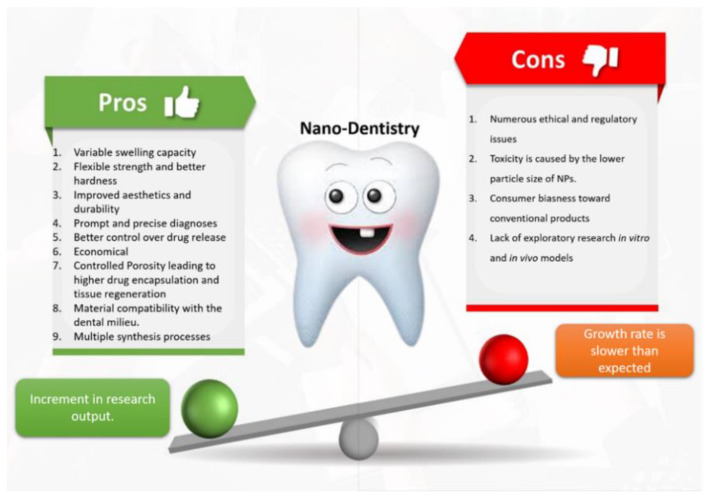
Visual presentation to demonstrate how advantages give thrust to nano dentistry research by tilting the balance towards the pro’s side.

A tectonic shift in the aim of nano dentistry was recorded with the advancements of the vast scientific research in the field of nanotechnology. The aim has now shifted from developing just a supportive nanocomposite to developing a highly efficacious bio-mimicking biomaterial for restorative dentistry. It was observed from the research data extracted from the Pubmed search platform that for a very long-time dental research was focused on nanocomposite only; however, with time, the research trend shifted towards other and more novel topics (Table 1), such as nanozymes. Meta-analysis of the previously published research abetted us to analyze the disadvantages and advantages of nanodentistry that were responsible for restrictive growth and relatively higher research trend in recent times, respectively. Figure 1 summarizes the advantages and disadvantages of nanodentistry.

**Figure 2:**
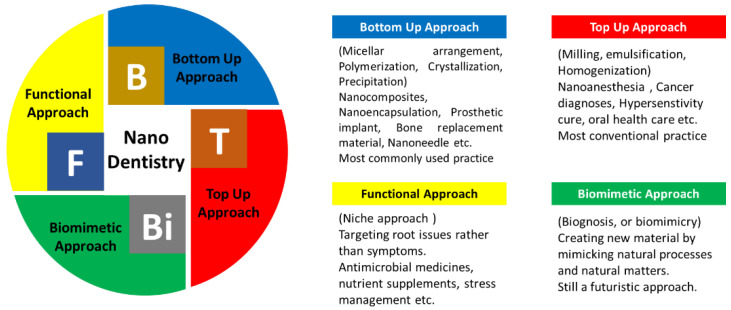
Various synthesis approaches implemented in nanodentistry.

It is already mentioned earlier that constructing a nano dental product is an intricate technique requiring matter and synthesis approach knowledge. These approaches were used to generate novel nanomaterials to synthesize regenerative material, implants, and drug enclosing nanoparticles (NPs), etc., [6]. Nanodentistry can be utilized to generate practically any product using any of the mentioned approaches. The never-ending tally of the nano-dentistry product includes nanocomposites, nanoimpressions, anti-infective mouth rinse, or the very commonly used nanofillers. Figure 2 illustrates the synthesis approach used in nanotechnology and how these approaches can be explored for manufacturing a tally of nano dental products. Where bottom-up and top-down approaches are the most commonly used practice for constructing nanodentistry products, the biomimetic approach is still in the research phase. Hence, in the present review, only those approaches that followed either the bottom-up or top-down approach were cited. 

**Figure 3:**
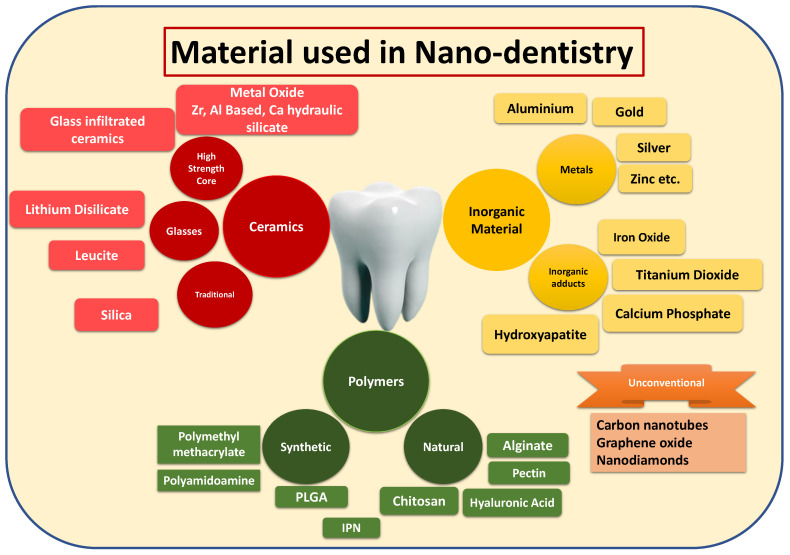
Diagrammatic illustration to provide basic material classes used in dentistry.

**Figure 4:**
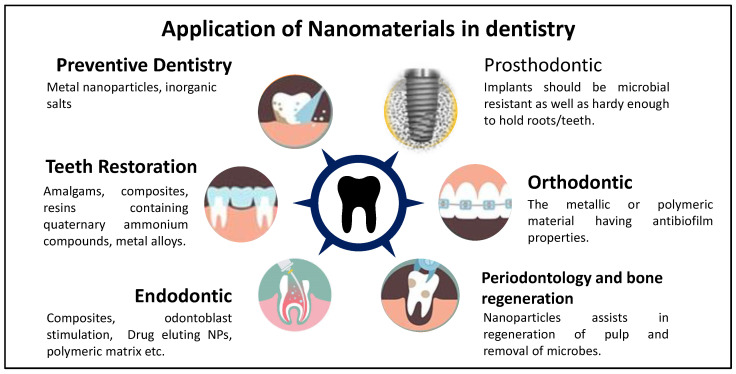
Pictorial representation of applications of nanomaterial in dentistry.

It is already mentioned that understanding the biomatters helps to make biomimicking NPs. This usage of biocompatible material leads to non-toxic NPs that can be retained at the physiological site for longer periods of time. Moreover, the material used in the construction of the dental product is different from other NPs due to changes in dental physiology, which is already being presented in detail in the previous section. Figure 3 demonstrates the different ways of usage of nanomaterial in nanodentistry. Dental biomaterials have been studied for decades. These can be classified primarily into four groups such as metals, ceramics (carbons, ceramic lenses, and glasses), metals, and polymers [7]. Polymers can further be divided into synthetic or natural products (both plant and animal products) [8]. With the emergence of eco-friendly and green chemistry, increased demand for natural polymers has been witnessed. Nanotechnology has broadened the holistic view of the usage of biomaterials in dentistry. Through the current review, we will present an intricate insight into how nanotechnology assists in designing newer and novel alternatives for different branches of dentistry (Figure 4).

## 2. Application of the Nano Dentistry

### 2.1. Preventive Dentistry

Preventive dentistry applies a functional approach to treatment, where the root cause of the symptoms is treated. Many metal NPs documented in past literature have shown enormous potential as antimicrobial in treating tooth decay or other dental ailments [8]. Dental experts also emphasize preventing tooth decay by inhibiting caries through controlling biofilm, enhancing remineralization, or providing antibacterial prevention to the oral cavity [9,10]. Hence, nanotechnologists also working on toes to provide novel and efficacious alternatives. Dental preventive products work by two means either by reducing the biofilm production or by restoring the damaged tooth by remineralization techniques to inhibit the further damage to the pulp and arresting the decline. Tooth decay begins with demineralization of the enamel owing to environmental changes. Changes in the microbial homeostasis in the buccal cavity lead to fermentation of the regular intake of sugars leading to acidification of the microenvironment, leading to the generation of biofilm, which through an intricate interaction with microbes, gradually dissolves the enamel, followed by the production of carries. If ignored further, that may infect the inner organic part of the tooth and lead to dentine and pulp decay, eventually the loss of teeth [11]. Preliminarily focus was to remove the deposited biofilm or inhibit the synthesis of biofilm by maintaining the lower oral pH level [12]. NPs high surface-area-to-volume ratios enable robust drug- or drug-combination loading that may result in synergistic antibiofilm efficacy [13]. However, smaller particle sizes, desirable charge, and hydrophilic/hydrophobic characters favor the protective film adhesion over the teeth surface. Fluoropolymer matrix coating layers are popular for their easy-to-clean purposes [14]. Being a theta surface (lower surface free energy), they easily allow detachment of microbial bilayer from the tooth surface under the influence of physiological shear. These coatings are particularly advised for patients with higher carries risk, i.e., xerostomia. It is evident that effective interaction between biomineral and bacteria is only possible if nano-sized particles that are smaller than the microorganisms are used [15].

The casein phosphor-peptides stabilize calcium and phosphate ions through the formation of amorphous calcium phosphates employed as biomimetic composites that diminish microbial adherence by attaching to the surfaces of microbial cells. Further antimicrobial use of metals has also been explored for centuries. Zinc oxide (ZnO) has been used in many conventional products for its antimicrobial properties [16]. Many kinds of toothpaste also incorporate Zn acetate and citrate to control the formation of plaque [17]. Previous literature demonstrated that particle size is inversely related to antimicrobial activity. Similarly, HA of the NPs also influences the microbial interaction with NPs [18]. Moreover, the literature suggests that bacteria are more likely to acquire resistance to conventional antibiotics viz a viz. NPs. [19]. Therefore, nanoparticulate metals were considered a better choice for exploration in antimicrobial activity. Nanosilver (Ag NPs) and copper have received a lot of attention due to their antimicrobial activity [20]. Recently, Ahmed et al. beautifully reviewed much recent research on the plant-based synthesis of Ag NPs [21]. Similarly, other green chemistry was also reported and reviewed by many researchers in different metallic NPs such as Zinc (Zn) and Nickle (Ni). [22,23]. Besides, plenty of other metals were found to play a dynamic role in preventive nanodentistry, such as copper and titanium. Ag salt NPs were precipitated to synthesize composites that release bactericidal Ag + ions in the case of Ag salt. Zhang et al. reported that the addition of nanoparticulate silver in quaternary ammonium dimethacrylate markedly reduced the microbial biofilm viability required for achieving better antibacterial activity [24]. Similarly, Chang et al. [24] also illustrated the use of Nano Ag with quaternary ammonium dimethacrylate in the ‘Scotch bond Multi-Purpose’ primer. The association provided the best outcomes, with augmented antimicrobial effect and attenuated lactic acid production. The same group also substantiated their previous findings by providing the combinational effect of Ag NPs, NPs of amorphous calcium phosphate (NACP), and quaternary ammonium methacrylates in controlling the biofilm [25]. Whereas Dias et al. investigated the effect of modification of Ag NPs with titanium dioxide on microbial biofilm and demonstrated a reduction in the microbial flora generation and attachment. Magalhães et al. compared the performance of three different marketing cementing, i.e., Sealapex, RelyX ARC, and Vitrebond, after incorporating Ag NPs. Inhibition halo was evident in siver NPs groups viz a viz naïve group where Ag NPs are not incorporated, indicating better antibacterial properties due to Ag NPs [26]. Gold is a conventional varnish material for protecting the teeth because of its excellent biocompatibility [27]. Zhang et al. investigated the role of antibacterial 4,6-diamino-2-pyrimidinethiol-modified gold NP-coated aligners on the bactericidal and antibiofilm efficacy against *P. gingivalis*. Results presented gold NP-associated aligners as an alternate treatment against such infections than the already present conventional periodontal treatment [28]. Recently, Kamath et al. developed gold NPs varnish for tooth remineralization and found positive outcomes [29]. There are many oral rinse products, such as mouthwashes and gargles, which are equipped with NPs for better efficacy. Similarly, in accordance with this, Kovtan et al. developed a paste or a rinsing solution containing calcium phosphate NPs, functionalized with the antibacterial agent chlorhexidine. Calcium carbonate, a natural physiological substance in the tooth, helps in the remineralization of the enamel loss due to microbes and serves as the second line of defense in controlling the decay. Moreover, chlorhexidine provides an antimicrobial effect in the oral microenvironment. There are plenty of mouthwashes available on the market that uses chlorhexidine as active pharmaceutical. However, scientists have offered a lucrative way to present chlorhexidine-loaded calcium salt NPs for its advanced benefits. These NPs, on the one hand, provide an antimicrobial/antibiofilm effect; on the other hand, calcium salt helps to close the open dentin tubules at the root surface, providing a remineralization effect to damaged teeth [30]. Similarly, Sun et al. developed a nano-CaF_2_ spray dried powder that is apparently an effective anticaries agent in increasing the labile F concentration in oral fluid and thus enhancing tooth remineralization. Iron is associated as the essential element for the formation of dentin. Moreover, it has a role in the discoloration of dentin. Liu et al. presented the theranostic potential of ferumoxytol iron oxide NPs against biofilms harboring *Streptococcus mutans* [31]. 

Enamel loss induces dentin to explore, making it hypersensitive to cold and heat. Modified silica NPs are used to treat dental hypersensitivity through remineralization to cease expanding decay. Other than HA, hydraulic calcium silicate (hCSCs) re-mineralizes demineralized dentin is another type of ‘bioceramic’ gaining popularity among dentists due to their higher strength, superior biologics, and good physiochemical properties [32]. Moreover, hCSCs can re-mineralize demineralized dentin hence aiding in cementing the tooth and arresting spreading infection to pulp. Zhang et al. presented nano-HA toothpaste for remineralization of teeth to arrest carries formation using an artificial carries model [33]. Similarly, Huang et al. illustrated a comparative study between nano-HA and sodium fluoride and substantiated the higher potential of nano-HA in remineralization [34]. Unfortunately, the product can penetrate only up to a small depth into the dentin tubules resulting in partial protection. Dendrimer type versatile and novel nanotherapeutics were also established for remineralization. Liang et al. investigated the remineralization effects of poly (amidoamine) (PAMAM) dendrimer plus a bonding agent with NACP in a cyclic artificial saliva/lactic acid environment. After 20 days, a significant increase in the dentin layer supported the raised hypothesis [35]. Liposomes have been vastly researched in the area of biofilm; Nguyen et al. demonstrated that polysaccharide (hyaluronic acids)-coated liposomes have a better ability to adhere to the tooth than the uncoated liposomes [36]. Infect, the cationic charged liposomes membrane (via attachment of pectin, Hyaluronic acid, etc.) assist in better adherence to tooth HA and thus retain enamel for longer periods of time [37]. Moreover, the hydrophilic coating further provides better stability of biofilms in the salivary microenvironment.

Besides, there are other drug delivery systems that have been vastly being explored for the same purpose. Table 2 illustrates previous literature reporting advanced drug delivery systems that have been considered prominent in controlling caries or helping in the restoration of the teeth.

### 2.2. Tooth Restoration

Following carries management, tooth restoration is the second most impeccable part of dentistry. Restoration is generally done by many methods starting from fillings, crowns, implants, bridges, and implants. From the nanotechnological point of view, fillers and implants are the most popular, as shown in Table 1. Nanofillers present a better adherence to teeth than the larger size fillers. Restorative filler materials used for remineralization of the tooth architecture can be classified according to their matrix component (ceramic, metal, polymer). However, these composites have their advantages and disadvantages. For example, ceramics composites are very brittle and polymeric composites are prone to high wear and tear. Literature reviews advocate the use of biological polymer/ biomimetic polymers to synthesize dental composites. 

HA is the main inorganic mineral component of teeth. Many scientific groups are working to synthesize different adducts of HA to form newer and novel nanocomposites, which may mimic the physiological elemental composition of teeth. For example, Chung et al. were successful in preparing a physiological component mixture by mixing NPs of HA (inorganic component: 75% w) with chitosan (25% w) to behave as an organic component of nanocomposites [65]. Sharifi et al. prepared HP–gelatin/curcumin nanocomposites and evaluated their antimicrobial effects against *Escherichia coli, Staphylococcus aureus*, and *Streptococcus mutans* [66]. There are various reports where the group has used different natural polymers for changing the properties of the organic component of the nanocomposites [67]. Consecutively, other scientific groups presented HA nanocomposites coupled with the antimicrobial effect of metallic NPs such as silver (Ag), gold, iron, silica, and Zn (Zn). There are literature where multiple metal usage augmented the efficacy of the product. Imani et al. developed HA/CuO/TiO_2_ nanocomposites with the highest antibacterial properties. Development was manifested with the assistance of the robust Quality By Design principle [68]. Recently, Balu et al. took a challenging task to accommodate Ag NPs, graphene oxide (GO), multi-walled carbon nanotubes (MWCNTs), and graphene oxide nano-ribbons (GONRs) in HA nanocomposites. The group studied the effect of the proportion of carbon on the final hardness of the nanocomposites. Moreover, the Ag NPs activity was evaluated using biostrain (*E. coli* and *S. aureus* bacteria). The developed nanocomposite provided a model drug release (lidocaine) that may benefit the patient [69]. Other synthetic polymers have been explored as an adjunct in nanocomposites preparation. Improvement of the mechanical properties of Poly Methyl Methacrylate Nanocomposites as nanocomposites [70] and compared with HA nanocomposites. Moreover, the drug (theobromine) eluting character of these nanocomposites was also examined [71]. 

Although, the research focus has shifted from analyzing the proportion to identifying and calibrating newer techniques to form nanocomposites. Hou et al. presented three sequential one port synthesis methods, where Ag ions are absorbed on the HA nanorods. The process is simple and validated using an array of pot synthesis methods of the nanocomposites. Whereas, Mallakpour et al. developed multiple component nanocomposites (Polyvinylpyrrolidone/L-leucine Amino Acid, Functionalized Mg-Substituted Fluorapatite Nanocomposites) employing ultrasonic waves as an energy source [72]. While various scientific groups used numerous other sources of energy, such as microwaves [73,74,75], photons [76] or their combination [77]. 

### 2.3. Endodontic

Due to higher vascularity, the pulp of the tooth is considered the most vital, fundamental life of the tooth. Hence it is very much important to restore it. Endodontics is the branch of dentistry that deals with the dental pulp and the area surrounding the dental pulp. It is needed when the infection crosses the upper part of the teeth, i.e., carriers and reaches to innermost part, i.e., the pulp. It deals with a specialized antimicrobial as well as constructive aids to provide long-lasting stability to teeth. However, pulpitis is a painful and irreversible process, requiring the removal of the entire pulp with a root canal and replacing the tissue with an inner polymeric material. However, in case invasion of the inflammation is just superficial, pulp regeneration is also possible. However, pulp regeneration is difficult and variable with respect to age. Dentin Odontoblast (present at the periphery of the pulp) stem cells have also been explored for the aforementioned purpose but illustrated a futile response in the clinical setup [78,79]. Hanafy et al. explored two commonly used dental biomaterials, i.e., mineral trioxide aggregate (MTA) and nano-HA, as odontogenic differentiation promotor results demonstrated significantly higher and upregulated expression of the characteristic genes for odontotomy differentiation, i.e., OPN, RUNX2, OCN, and Collagen1, in the case of treatment group than the control [80]. Pulp capping is another method to preserve the vitality of the pulp and contain the infection outside the vascular region. Li et al. proposed biocompatible, osteogenesis-sensitizing properties and a combination of micro-nano bioactive glasses. Ca-Zn-Si-based (Zn doped) bioglasses micro nanospheres for dental pulp capping. Results were encouraging to show higher antibacterial effects and higher stimulation of macrophages to reduce the proinflammatory markers, followed by remineralization of dentin via sensitization of dental pulp cells [81]. Sinjari et al. proposed a very advanced nanotechnological approach using drug encapsulated liposomes to reconstruct the homeostasis of dental pulp stem cells. The treatment was able to restore cell proliferation and attenuate the inflammation markers hence aiding in tooth restoration regarding 2-hydroxyethyl methacrylate [82]. Similarly, Kim et al. proposed an RGD peptide conjugated dendrimer-based drug delivery system for dental pulp differentiation after a traumatic dental injury. Results were very encouraging, with higher mineralization and odontogenic potential [83]. Elgendy et al. explored natural scaffolds, i.e., propolis, chitosan, for tooth restoration with a very limited set of experiments and provided their potential for endodontic treatment due to elevated biocompatibility and tissue restoration ability [84]. In the same line, Tondnevis et al. proposed the development of a dental tissue scaffold containing nano-HA or Nano fluoro HA /Chitosan scaffold using polymers using the freeze-drying technique. Results illustrated that the addition of chitosan assisted in significant increases in cell proliferation [85] These studies illustrated the potential of chitosan NPs in dental endodontics. Recently a laboratory study was reported by Bhaskar et al. dictating the role of eggshell-derived porous nano-HA and CMC (Carboxy Methylcellulose) composite and showing their effect on dental bioactivity and cell proliferation using the significant elevation of VEGF and dentine sialophosphoprotein [86]. Amoxicillin-loaded nanodiamond Guttapercha composite (NDGP-AMC) was developed and tested for its use in root canal treatments with promising results [87]. Other than these other natural fibers and polymers, i.e., gelatin [88,89,90], collagen [91,92], and silk [90] have been explored in nanodentistry. 

### 2.4. Prosthodontic

With the advent of technology, living standards have drastically improved attitudes towards oral health. Therefore, interest in prosthodontics for advancing research on artificial restorative materials has been shifted to the upside [93]. Classically, these materials are broadly categorized into ceramics, resins, polymers, and metals [94]. However, a few issues are still unsolved to cater to the need for prosthodontics. Here, nanotechnology played a vital role and different nanomaterials have been synthesized/utilized to cover the untapped areas of prosthodontics. Nanotechnology has also significantly improved the properties of existing materials, such as ceramics, impression materials, denture bases, and types of cement used in prosthodontics. 

Prosthodontics are mainly classified into removal or fixed type (tooth-supported or implant-supported)

#### 2.4.1. Removable Type

In this category, Polymethylmethacrylate (PMMA) polymers and silicone elastomers are mainly used for denture and maxillofacial prostheses, respectively. However, dimensional changes with PMMA polymers and poor tensile and tearing strength of silicone elastomers pave the way for using nanotechnology in these types of restorative materials [95]. Firstly, carbon nanotubes lead to a bond formation with PMMA resins/polymers by weak van der Waal force to improve its dimensional stability for better fit and tensile strength [96]. Secondly, metal-based NPs (Ag, TiO_2_, etc.) reinforce PMMA polymers to improve their tensile strength and antimicrobial activity [97]. Moreover, polyhedral oligomeric silsesquioxanes (POS), a nano (1.5 nm) silica cage, and metal NPs support silicone elastomers to improve their tensile strength and physical properties [98,99]. Moreover, nanotechnology improved the pathological states like denture stomatitis induced by adherence of biofilm of *Candida albicans* over the denture base materials [100]. Moreover, Alumina NPs were synthesized and evaluated for improving osseointegrity and curative process when the dental implant was installed [101].

#### 2.4.2. Fixed Type

In this category, tooth and implant-supported nanomaterials, nanocomposites, and nanocoatings are primarily used as fixed prostheses. The issue of dimensional unfolding persisted in the conventional fixed types of restorative materials. Conventional materials also showed cytotoxic effects by leaching organic monomers into surrounding gum tissues [102]. Nanocomposites based on nanofiller technology solve these issues [103]. Silica or Zirconia-silica NPs were synthesized by treating silica particles with 3-methacryloxypropyl-trimethoxysilane to improve flexural strength and hardness. Nanotechnology also brings hope and new vistas in improving the adhesion and durability of implants. Carbon nanotubes, polyvinyl alcohol, and silica-based NPs were used in addition to calcium phosphate to synthesize nanocomposites and sometimes scaffolds for improving mechanical strength and tissue regeneration [104]. In addition, the improvement of calcium carbonate-silicone dioxide NPs improves tear strength and hardness of maxillofacial silicone elastomers [105]. In another study, Persson et al. synthesized glass-ceramic in Zirconia-silica NPs using the sol-gel method to improve the corrosion resistance and hardness [106].

Few commercial products are also available in the market to understand the potential of nanotechnology in prosthodontics. Clearfil Majesty Posterior is a product (a superfilled nanohybrid composite) of Kuraray America and is an ideal amalgam alternative (92% filled with nano-sized alumina and glass materials) and shows high physical properties with superior surface wear resistance [107]. Premise^TM^, a product of Kerr corporation (84% filled), a nanocomposite that shows significant improvement in durability, wettability, and sculptability [108].

### 2.5. Orthodontics

Orthodontic brackets and archwires are important components for desired tooth movement. However, it may take time to acquire better holding and stability due to frictional forces between orthodontic wires and brackets [109]. To trace these issues, coating these components using nanotechnology is used. Nowadays, Fullerene like Molybdenum and tungsten disulfide NPs are used as dry (solid) lubricants for stainless steel archwires to reduce the friction [110]. Moreover, it was found that maintaining oral hygiene is more difficult during orthodontic therapy due to microbial colonization (e.g., *Streptococcus mutans, Staphylococcus aureus,* etc.), a biofilm of bacterial plaque and enamel decalcification. Several studies have been reported using nanotechnology-based approaches like elastomeric ligatured supported NPs (Benzocaine and Ag) to show anticariogenic, antimicrobial activity [111] and nanocomposites (Ag NPs with ZnO, chlorhexidine) of adhesive types/bands to elicit improved tensile strength, anti-inflammatory properties [112]. Akarajarasrod et al. reported improved antibacterial activities of orthodontic adhesives when incorporated with gold NPs [113]. In a study, a comparative evaluation between orthodontic adhesive containing silver NPs and conventional orthodontic adhesives was reported by Ahn et al. This study illustrates that newer orthodontic adhesives prevent enamel demineralization better than conventional adhesives [114]. Another study showed that the inclusion of TiO_2_ NPs in orthodontic composites improved the antibacterial activity without losing the integrity of shear bond strength [115]. Moreover, it was found that copper material was more stable (physically or chemically) and economical than silver material. Eshed et al. also showed a significant inhibitor effect when copper oxide NPs were incorporated with adhesive [116,117].

Further, nanotechnology brings new hope to orthodontic therapy by utilizing nanocoating over temporary anchorage devices, especially mini-screws, for improving wettability and anti-inflammatory activities [118]. Overall, the use of nanotechnology in orthodontics and prosthetics opens the door to reducing/avoidance of demineralization and shows more anchorage leads to improved patient compliance [119].

### 2.6. Periodontology and Bone Regeneration

Due to several factors like genetics, smoking, poor oral hygiene, and obesity, periodontitis occurs, leading to the destruction of the tooth and its surrounding structure (including the cementum, alveolar bone, and ligament). Therefore, the overall aim of periodontal therapeutics is to repair or for bone regeneration [120]. Several aspects have been studied but found shortcomings. Nanotechnology has also gained popularity in this field, reducing dentin hypersensitivity, drug delivery, and bone regeneration.

Dentinal hypersensitivity is one of the major concerns patients face in periodontal diseases. HA NPs (HA NPs) were studied and clinically used in toothpaste and mouth wash (nano-XIM of FLUIDINOVA, S.A. Portugal) as these particles are highly effective in sealing dentin tubules and preventing nerve revelation. These NPs also showed higher binding to teeth surface, thus promoting enamel remineralization [121,122]. Moreover, drug-loaded NPs and scaffolds are also interestingly popular for tissue regeneration and healing periodontal diseases [123]. Various biodegradable polymers like Poly(Lactic acid-Glycolic acid) (PLGA) based NPs were successfully formulated to deliver therapeutic actives like minocycline to show sustained release patterns in periodontal infections [124]. HA-NPs loaded tetracycline were also synthesized and promoted the proliferation of periodontal ligament cells [125]. Further, tetracycline NPs have also been investigated for periodontal treatment as well. There are various commercially available tetracycline-loaded microsphere patches, such as Arestin^®^ (Valeant, Bridgewater, MA, USA) and Nanogen^®^ (Orthogen, Springfield, IL, USA), already in the market. These are intended for insertion within the periodontal pocket, which releases the drug into the affected area in a sustained manner. Various growth factors (e.g., Bone Morphogenetic Protein-2 and Fibroblast) loaded NPs were synthesized to initiate the alveolar bone cell differentiation process [126]. Furthermore, various scaffolds based on nano-engineering exhibited potential in bone repair due to mimic of native tissue construct. Various electrospun nanofibres (prepared by PLGA or gelatin) were utilized to grow the human periodontal ligament (PDL) cells by exhibiting the optimum cell adhesion and differentiation process [127]. Also, the scaffolds containing nano-HA and nano-alginate were prepared to culture human PDL cells [128]. Besides polymeric nanofibers, various biomimetic self-assembling peptides (e.g., P_11_-4) were also studied and evaluated to treat early caries lesions [129]. Moreover, green chemistry-based biomaterials were also invented and utilized for oral bone regeneration. Gandolfi et al. formulated a hydrogel consisting of polysaccharides (copolymers of L-guluronate and D-mannuronate) and peptide (gelatin) matrix activated with biominerals (calcium silicate, dicalcium phosphate dehydrate) type of fillers and evaluated this green hydrogel for bone regeneration [130]. Also, they had developed Poly-lactic acid (PLA) based scaffolds loaded in exosomes with suitable minerals (calcium silicate and dicalcium phosphate dehydrate; DCPD) to improve the osteogenic strength of human adipose-derived mesenchymal stem cells [131]. In another study, Tatullo et al. developed an innovative scaffold for regeneration of periapical and alveolar bones using polymeric (PLA) base doped with minerals (hydraulic calcium silicate and DCPD) seeded with human mesenchymal stem cells of the inflammatory periapical cyst. These scaffolds were produced by thermally induced phase separation technique, whereas other techniques are also utilized to produce polymeric scaffolds: electrospinning, salt leaching, and 3D printing [132]. Further, many porous nano-scaffold have been in use for tooth extraction site preservation. Nano-HA mineralized silk fibroin porous scaffold is biocompatible in nature and helps in bone regeneration. The porous nature simulated the biological bone structure and induced osteogenesis in in vitro cell line culture [133].

### 2.7. Dental Implants

For many decades, dental implants have been used and applied to restore or replace teeth. However, attaining osseointegration and managing infection associated with implants (metallic type) are still an issue to explore [134]. Various studies have been reported for using different surface modifications to improve these issues, as surface modification also affects the biological regenerative process (adsorption of proteins, adhesion, differentiation, and proliferation of cells, which further initiates tissue regeneration). Nanotechnology-based coating over the surface of titanium and tetragonal Zirconia implants was studied to improve the biological and mechanical properties [135]. Further, Silica nanocomposites were utilized and coated over the surface of implants to improve mechanical strength and bone growth [136]. Moreover, the implant surface can be treated/modified by HA nanocrystals [137] and Calcium-phosphorus NPs for ameliorating bone regeneration [138]. In addition to the advantages of metal and ceramic-based dental implants, several limitations like toxicities and corrosive behavior are also associated with them. To overcome these challenges, less toxic plant-derived biomaterials are used in the production of dental implants [139]. A recent study reported that gold NPs were photo-fabricated from aqueous bark extract of *Salacia chinensis* and applied in dental implants, where it was shown its biocompatibility with blood and high osseoinductive potential when evaluating the cell viability with MG-63 bone osteosarcoma cell lines [140]. Another approach to improving osseointegrity of dental implants is the use of calcium phosphate nanoparticles coated over the surface of TiO_2_ implants [141]. In another study of rats with ovariectomized induced osteoporosis, where chitosan gold NPs carrying the c-myb gene improved the osseointegration of dental implants [142].

### 2.8. Regulatory Framework

Although nanomaterials have solved various challenges associated with dentistry. However, various concerns such as toxicity and regulations are not solved fully and need to be understood to explore nanotechnology potential fully. In comparison to the conventional materials/formulations in dentistry, nanomaterials show different responses due to their unique physiochemical properties such as size, interaction with different cell membranes and other biological macromolecules, and different pharmacokinetics parameters. Therefore, more understanding of these interactions, toxicity assessment (especially immunotoxicology and impurity profiling), in vitro–in vivo modeling, and regulations are necessary to increase the commercialization success rate of nanomaterials, especially in dentistry [143].

NPs induced toxicity symptoms can be easily understood by different nanomaterials utilized in dentistry. Carbon nanotubes used in dental coating showed some inflammatory reactions when in contact with some biological membrane [144]. Moreover, during the preparation of nanomaterials, exposure to them by inhalation may cause toxic symptoms in the lungs. Exposure to titanium dioxide (TiO_2_) NPs may impose a risk of lung cancer [145] and impair spatial cognitive performance [146]. HA NPs employed in a dental filling bind with blood proteins, form a complex that is killed by macrophages, and then transferred to major vascular organs, such as the lung and spleen, which shows toxic responses [147]. In addition, genotoxic and immunotoxic effects were exhibited by silica and zirconium nanomaterials primarily due to reactive oxygen species (ROS) [148,149].

Unique physio-chemical properties, surface charge, and the high surface-to-volume ratio of NPs act as a basis for transporting them into the cells to illustrate toxic effects. Different scientific reports revealed the internalization process of NPs in the mammalian cells and expressed the endocytosis as a transport mechanism through clathrin mediation and scavenging receptors. However, the interactions of nanomaterials with alveolar bone and gingival mucosa are still unknown and need to be explored clinically [150]. Moreover, the presumed mechanism for toxicity is the induction of cellular oxidative leading to more production of ROS, which further leads to altering cell signaling cascades, generating protein radicals and lipid peroxidation, DNA damage, initiation of inflammatory responses, and eventually cell death [151]. Recently, metals in dentistry have raised another challenge and concern for environmentalists. Grinding of resin-based dental materials results in wastewater pollution, especially in the case of using micro-nano particles making their way from dental sinks to the wider environment leading to potential toxicity concerns [152]. Especially after the ‘Minamata convention,’ the use of mercury amalgam is being replaced with resin formulation in dentistry [153]. However, scientists present the solution for remediation of heavy metal poisoning in waste dental water using NPs only. These specified nano pollution absorbers and efficiently eradicate heavy metals from water due to their higher surface area and porous nature [154].y 

In a nutshell, to fasten the regulatory submissions and commercialization of nanomaterials in dentistry, more understanding of material selection (whether it is biocompatible and biodegradable or not), particle size, surface charge, and interaction with biological membranes are required.

## 3. Future of Nanodentisty

Undoubtedly nanodentistry confers numerous advantages over conventional systems, such as higher bio-regeneration, a notable antimicrobial effect due to anti-biofilm properties, increased hardness of composites, and better sealing of fillers, but at the same time, its overpricing, precise placement, associated toxicity, costly development, and international regulations limit the clinical exploration. Though despite all the stated hurdles, scientists are now working hard to find the least expensive methods to synthesize NP scaffolds that fits in the regulatory framework as well as assist in placing the NPs into the right place. There are multiple unconventional NPs, including nanodiamonds, quantum dots, nanoshells, and carbon nanotubes, which have been explored widely in research and have better outcomes to be used in future commercial markets. Its superior surface and chemical nature make it a very suitable candidate for use as a filler in dental nanocomposite fabrication. 

### 3.1. 3D Printing

3D printing is one technique that is being employed for the synthesis of the most complex gematrical scaffold that might be difficult to make when using different processes. Chau et al. fabricated vancomycin releasing polycaprolactone/nHA nanocomposite using 3D modeling. The scaffolds showed higher strength and sustained drug release for up to 14 days which may assist tissue regeneration with antimicrobial activity [155]. However, despite the success in bone regeneration scaffold synthesis, little research has been done in the nanodentistry domain, making it a potential area for future research.

### 3.2. Nanobots

To overcome the already stated shortcomings, i.e., precise placement, nanorobots have been invented. These are specialized tools to carry out programmed penetrations, clean the decayed tissue, and place composites to the required site using 3D filling technology. Dasgupta et al. published the use of magnetic nanobots incorporated in root canal operations. These nanobots could go deeper into the dentin, which is difficult using conventional methods. The special retrieval process of nanobots also made a better choice [156]. These nanobots work using a special algorithm or software. In 2015, Razavi et al. demonstrated simulation for dental restoration. The inclusion of robotics increases the speed by eight times [157].

### 3.3. Nanozymes

Inorganic NPs showing enzyme-like properties are termed nanozymes. They are less costly, easy to synthesize, more stable, and highly efficient compared to their natural counterparts. They are vastly used for their theranostic applications. Zhang et al. developed DNA nanozymes for biosensing the presence of dental bacteria [158]. Similarly, Huang et al. presented bifunctional nanozymes that specifically inhibit the pathogenic *Streptococcus mutans* (pathogen) but not the commensal, *Streptococcus oralis* using iron oxide nanozymes having oxidase kind activity [159]. 

## 4. Conclusions

Conventional dentistry is enriched by revolutionized nanotechnology despite the many irrefutable lacunas that limit its clinical exploration. Research in the field of nanodentistry is still lagging as compared to other biological research areas. More patient-centered research will help to boost the development of nanotheranostics that should not only be efficacious but, at the same time should be affordable. Moreover, the true potential has still not been unleashed in the case of nanodentistry until now; metallic/polymeric NPs have only been explored vastly, but drug-eluting NPs research still has gigantic leaps to follow. Although challenges are huge, a collaborative scientific effort will make the impossible possible.

## Figures and Tables

**Table 1:** Research topic click number in PubMed.

Year	Nanotechnology in Dental	Nanodentistry	Nanoparticle in Dentistry	Nano Implant	Nanorobot	Nano Composites
2022	3	1	6	2	0	24
2021	182	5	513	84	1	2300
2020	154	5	475	74	0	2266
2019	99	0	385	61	2	1934
2018	108	5	343	68	3	1901
2017	102	0	297	63	0	1810
2016	97	5	277	57	0	1822
2015	89	3	251	60	3	1822
2014	84	5	210	42	2	1705
2013	52	3	148	35	1	1679
2012	49	3	127	32	0	1638
2011	64	3	91	31	2	1451
2010	42	1	73	21	1	1359
2009	42	0	62	23	0	1301
2008	25	1	58	11	0	1301
2007	40	0	44	6	0	1247
2006	35	0	12	4	1	1189
2005	24	0	11	7	0	1109
2004	22	0	5	2	0	998
2003	18	0	2	0	0	914
2002	5	0	2	1	0	838

**Table 2:** Previous reports on various prominent advanced drug delivery systems show significant contributions to nanodentistry.

Drug Delivery System	Characteristic	Action	Reference
Dendrimer	Nitric oxide-releasing alkyl-modified poly (amidoamine) dendrimers	Antibiofilm against *Streptococcus mutans*	[38]
	poly (amidoamine) containing amorphous calcium phosphate NPs	Remineralization	[39]
	Chlorhexidine encapsulated PAMAM dendrimer	Remineralization in etched human dentin in vitro	[40]
	PAMAM dendrimer containing nitric oxide	Antibacterial antibiofilm effect	[41]
	Alendronate conjugated dendrimer targeting hydroxyapatide (HA).	Alendronate targeted the PAMAM to HA and cooh-PAMAM induced HA crystallization.	[42]
	Anionic PAMAM dendrimer in remineralization	De-mineralized human enamel is mineralized in perfectly arranged rod-like crystals.	[42]
Solid Lipid NPs	Mentha spp. Essential oil encapsulated solid lipid NPs	Antimicrobial potential helps in reducing the carries	[43]
Chitosan NPs	Miswak, chitosan NPs, and propolis as varnishes	Anticaries action using antibacterial effect against Streptococcus mutans (S. mutans)	[44]
	Rutin (a flavonoid) encapsulated chitosan NPs	Antimicrobial effect against *Bacillus pumilus* and *Enterococcus faecalis*, Anticariogenic potential	[45]
	Hybrid copper-chitosan NPs (Core of Copper with chitosan envelope)	Beneficial to controlling dental plaque formation	[46]
	CuO NPs capped with chitosan	Works as a binding agent and provides a remedy for secondary caries.	[47]
Ag NPs	Combination of ethyl methacrylate with Ag ethylhexanoate	Improved the mechanical properties of composite and efficacy of the product increased in terms of reduced biofilm production	[48]
	Ag NPs	Antimicrobial effect was seen over teeth using an in vivo experiment	[49]
	Synthesis of Ag NPs using locust bean gum (LBG) polysaccharide plant extract.	Effect of pH was noticed on the size and stability of NPs	[50]
	Synthesis of Ag NPs using fungal	By modulating the temperature	[51]
	One-pot biosynthesis using marine sponge (*Haliclona exigua*) for the synthesis of flower-like Ag NPs	Significant antiproliferative activity and antibacterial activity evident in biofilm bacteria	[52]
	Characterize and evaluate the stability and toxicity with re-mineralizing effects of silver NPs and fluoride anticaries agent (AgF) on staining dental enamel.	The product represented long-term stability, superficial and in-depth remineralizing capacity with antimicrobial potential and biocompatibility and did not stain the enamel.	[53]
	Implant survival rate has been increased by incorporating Ag porous NPs in polymer matrices of polycaprolactone/polyvinyl alcohol on titanium implant	Ag NPs adsorption on the surface causes the surface to have a porous structure. And provide better biocompatibility, antimicrobial activity, etc.	[54]
Zinc (Zn)	Functional remineralization of dentin effect of Zn-doped polymeric NPs	Clinical outcomes as remineralization of teeth with antimicrobial properties as anti-biofilm.	[55]
	Impact of phyto-synthesized Zn Oxide NPs (ZnO NPs) using *Murraya paniculata* leaf extracts.	The NPs show anti-bacterial properties and can be used for dental composite, etc.	[56]
	Fabrication of composites cellulose/polypyrrole composed with ZnO NPs	Field Emission Scanning Electron Microscopic images demonstrated that bacterial cellulose structure was preserved with the addition of other agents and the resultant composites showed good antibacterial properties.	[57]
	Uniform Zn doped mesoporous NPs were evaluated for antibacterial effects	The mechanical property of composite is increased with is its bacterial effect.	[58]
	ZnO NPs were evaluated for their antibacterial coating over implants	ZnO NPs with HA NPs have shown increased antibacterial activity with osteogenic activity. Thus favoring tissue regeneration	[59]
Zirconium (Zr)	Effects of incorporation of Zr oxide NPs on antibiofilm activity, glucose sorption, weight change, and surface roughness of two different types of denture liners	The addition of 0.5% Zr oxide NPs made the dentures softer and provided significant anti-biofilm activity.	[60]
	Preparation of Zr NPs using NPs using Punica granatum (pomegranate) peel extract and evaluated for their antibacterial effect.	Antibacterial and antioxidant effect was evident, which can be explored for dentistry.	[61]
	Studied effect of zirconia NPs on mechanical properties of adhesive systems	NPs of ~50 nm shown adhesive layer or in primer increased tensile strength and promoted mineralization	[62]
Miscellaneous	A simple chemical method was used for the formulation of bimetallic Copper–Nickle (Cu-Ni) NPs and were evaluated for their antibacterial activity on human pathogens	Results were conclusive in giving better outcomes in antibacterial activity against Staphylococcus aureus (gram-negative) and Escherichia coli (gram-positive).	[63]
	Mechanophysical and biological characteristics of therapeutic cement after addition of Cu NPs	Cu in Zn phosphate cement showed significantly augmented odontoblastic differentiation using dental pulp human cells.	[64]

## Data Availability

Not applicable.
